# Treatment utilization and outcomes in elderly patients with locally advanced esophageal carcinoma: a review of the National Cancer Database

**DOI:** 10.1002/cam4.1250

**Published:** 2017-11-15

**Authors:** Gregory Vlacich, Pamela P. Samson, Stephanie M. Perkins, Michael C. Roach, Parag J. Parikh, Jeffrey D. Bradley, A. Craig Lockhart, Varun Puri, Bryan F. Meyers, Benjamin Kozower, Cliff G. Robinson

**Affiliations:** ^1^ Department of Radiation Oncology Washington University St. Louis Missouri; ^2^ Department of Medicine Division of Oncology Washington University St. Louis Missouri; ^3^ Department of Surgery Division of Cardiothoracic Surgery Washington University St. Louis Missouri

**Keywords:** Definitive chemoradiation, elderly, locally advanced esophageal cancer, palliative treatment, trimodality

## Abstract

For elderly patients with locally advanced esophageal cancer, therapeutic approaches and outcomes in a modern cohort are not well characterized. Patients ≥70 years old with clinical stage II and III esophageal cancer diagnosed between 1998 and 2012 were identified from the National Cancer Database and stratified based on treatment type. Variables associated with treatment utilization were evaluated using logistic regression and survival evaluated using Cox proportional hazards analysis. Propensity matching (1:1) was performed to help account for selection bias. A total of 21,593 patients were identified. Median and maximum ages were 77 and 90, respectively. Treatment included palliative therapy (24.3%), chemoradiation (37.1%), trimodality therapy (10.0%), esophagectomy alone (5.6%), or no therapy (12.9%). Age ≥80 (OR 0.73), female gender (OR 0.81), Charlson–Deyo comorbidity score ≥2 (OR 0.82), and high‐volume centers (OR 0.83) were associated with a decreased likelihood of palliative therapy versus no treatment. Age ≥80 (OR 0.79) and Clinical Stage III (OR 0.33) were associated with a decreased likelihood, while adenocarcinoma histology (OR 1.33) and nonacademic cancer centers (OR 3.9), an increased likelihood of esophagectomy alone compared to definitive chemoradiation. Age ≥80 (OR 0.15), female gender (OR 0.80), and non‐Caucasian race (OR 0.63) were associated with a decreased likelihood, while adenocarcinoma histology (OR 2.10) and high‐volume centers (OR 2.34), an increased likelihood of trimodality therapy compared to definitive chemoradiation. Each treatment type demonstrated improved survival compared to no therapy: palliative treatment (HR 0.49) to trimodality therapy (HR 0.25) with significance between all groups. Any therapy, including palliative care, was associated with improved survival; however, subsets of elderly patients with locally advanced esophageal cancer are less likely to receive aggressive therapy. Care should be taken to not unnecessarily deprive these individuals of treatment that may improve survival.

## Introduction

Esophageal cancer comprises a significant portion of gastrointestinal malignancies with an annual incidence of approximately 17,000 in the United States and over 450,000 worldwide 1, 2. Despite improvements in outcome over time, annual death rates continue to nearly match annual incidence and prognosis remains poor with an overall survival of around 20% at 5 years for locoregional disease 1, 2. Surgical resection has been a key component of definitive therapy; however, its utilization is often tempered by potential morbidity and mortality associated with esophagectomy 3, 4, 5. For surgically fit patients, a standard of care for locally advanced disease is trimodality therapy with neoadjuvant concurrent chemoradiation followed by esophagectomy based on improved outcomes compared to surgery alone 6, 7.

When aggressive combined‐modality therapy is a standard treatment approach as it is for esophageal cancer, the elderly patient in particular presents a therapeutic challenge. The decision whether to offer definitive therapy, or any therapy at all, is determined in the context of an increased risk of toxicity and a more limited life expectancy. Studies examining patterns or outcomes of treatment for esophageal cancer in this population are limited, and most are small, single‐institution experiences. In this setting, esophagectomy in the elderly is less utilized and associated with a potential increase in postoperative complications, but is feasible in selected patients with acceptable outcomes 5, 8, 9, 10. Definitive chemoradiation is also more infrequently considered in the elderly, yet treatment appears to be well tolerated with efficacy comparable to younger patients 11, 12, 13, 14. Population‐based analyses have elucidated some broad trends and outcomes in this older population and these largely mirror the single‐institution studies 15, 16. However, these studies predate the definitive treatment paradigm shift to neoadjuvant therapy and thus may be less applicable to a modern cohort of elderly patients.

To address current trends and predictors of treatment utilization and associated outcomes, we queried the National Cancer Database (NCDB). A joint program of the Commission on Cancer of the American College of Surgeons and the American Cancer Society, the NCDB compiles data from over 1500 commission‐accredited cancer programs and captures about 70% of newly diagnosed patients with cancer in the United States annually. Here, we examine elderly patients (≥70 years old) with locally advanced esophageal cancer to evaluate how this population is being managed in the modern era and what factors influence specific treatment choices. Modalities evaluated were no treatment delivered, palliative therapies, definitive chemoradiation, esophagectomy alone, and trimodality, with the primary aim to compare overall survival between these groups. Secondary aims included identifying variables independently associated with receiving each modality.

## Material and Methods

### Patients

The NCDB Participant User File for esophageal cancer was used to identify clinical stage II and III patients ≥70 years old diagnosed between 1998 and 2012. As patients and centers are deidentified by the NCDB, this study was deemed exempt by the Washington University School of Medicine Institutional Review Board.

Using the NCDB data dictionary, patient characteristics were dichotomized into: Caucasian or non‐Caucasian; population type of metropolitan, urban, or rural; average income <$38,000 or ≥$38,000 by zip code; education level of ≥21% or <21% in zip code with no high school diploma; and insurance status of Medicare, Medicaid, private, or other government (e.g., Federal insurance or Tricare). Using ICD‐0‐3 codes, histology subtypes were dichotomized as either adenocarcinoma or squamous cell of the esophagus.

**Figure 1:**
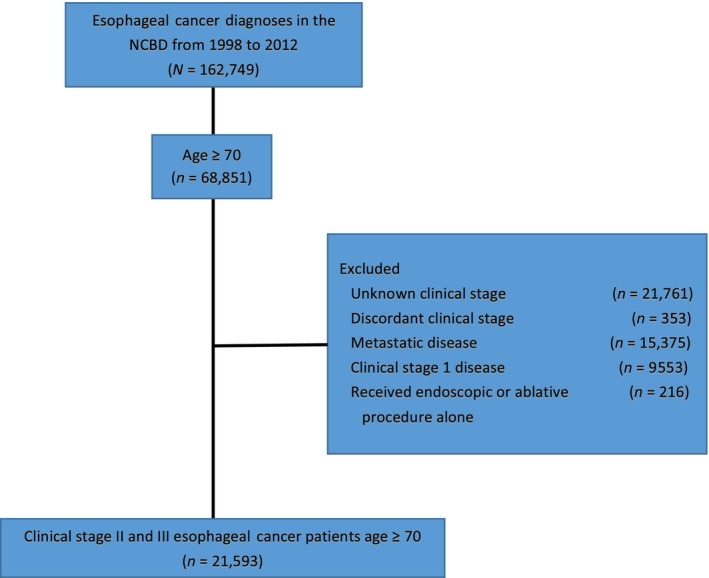
Consort diagram.

Patients were excluded from analysis if they had unknown clinical stage, discordant clinical stage (recorded clinical T/N stage did not match recorded overall clinical stage), metastatic disease, or received endoscopic or ablative therapy. A Consolidated Standards of Reporting Trials (CONSORT) diagram is shown in Figure 1. An interquartile range of center volume by the number of years a center contributed to the NCDB was calculated. Centers in the top quartile were labeled “high‐volume” centers, all others were labeled “low‐volume” centers. Patients were considered to have received concurrent chemoradiation if the date from diagnosis to initiation of systemic therapy and the date from diagnosis to initiation of radiation therapy were within a 14‐day limit. If these dates were separated by >14 days, they were considered to have received sequential therapy. For the purposes of this analysis, palliative therapy was defined as *any* treatment not considered definitive therapy for curative intent for locally advanced disease and included: chemotherapy only, radiation therapy only, or sequential chemotherapy/radiation therapy. Trimodality therapy was defined as receipt of neoadjuvant chemoradiation therapy followed by esophagectomy.

### Statistical analysis

Descriptive statistics of continuous variables were expressed as mean ± standard error of the mean. Univariate comparisons included independent sample *t*‐tests to compare normally distributed continuous variables and chi‐square analysis for comparisons of categorical data. Stepwise backwards logistic regression was used to identify variables independently associated with receiving palliative therapy (relative to no treatment) and trimodality therapy (relative to definitive chemoradiation) as this method allows for more robust analysis to determine significance in a large cohort. Criteria for entry into the logistic regression model included a *P* value of <0.05 on univariate analysis. Kaplan–Meier analysis was performed to compare median overall survival outcomes by therapy type (including no treatment), with log‐rank testing. A Cox proportional hazards model was created to identify variables independently associated with increased risk of overall mortality.

To assist in controlling for patient and tumor factors potentially involved in selection bias, two propensity‐matched analyses were performed: (1) matching no treatment patients to those receiving palliative treatment, (2) matching definitive chemoradiation patients to those receiving trimodality therapy, (3) matching definitive chemoradiation patients to those receiving esophagectomy alone. Patients were matched on the following variables: age (as a continuous variable, to better account for possible unmeasured age‐related comorbidities), sex, race, distance from treatment center, center type, center volume, insurance type, income, education level, population type, Charlson‐Deyo score, histology type, clinical stage, T stage, tumor grade, and tumor location. After propensity score calculation using logistic regression, patients were matched 1:1 using nearest neighbor matching with a caliper distance of 0.20 of the standard deviation of the logit of the propensity score. Postmatching diagnostics included analysis of the standardized mean differences between the matching variables. For both matched analyses performed, no interactions demonstrated a standardized mean difference >0.20. Kaplan–Meier analysis was again performed among these matched pairs with log‐rank testing.

*P* values <0.05 were considered statistically significant. Statistical analyses were performed in SPSS 23.0 for Windows (SPSS Inc, Chicago IL, 2013).

## Results

### Patient characteristics

A total of 21,593 patients with locally advanced esophageal cancer age ≥70 were analyzed (Table 1). The median age was 77 and patients were predominantly Caucasian (89.0%), male (72.0%), and of a higher educational (82.1%) and income (79.9%) status. There was a near‐even split between clinical stage II (52.6%) and stage III (47.4%) patients. The majority of tumors were located in the lower third of the esophagus (56.7%) and were of adenocarcinoma histology (52.1%).

**Table 1:** Demographics and clinical characteristics for elderly clinical stage II and III esophageal cancer patients from the NCDB diagnosed between 1998 and 2012

Demographic or clinical characteristic	Patients (*N* = 21,593)
Age at diagnosis, years (median)	77 (70–90)
Sex	
Male	15,544 (72.0%)
Female	6049 (28.0%)
Race	
Caucasian	19,227 (89.0%)
Non‐Caucasian	2366 (11.0%)
Charlson/Deyo comorbidity score	
0	11,413 (52.9%)
1	3440 (15.9%)
≥2	1119 (5.2%)
Missing	5621 (26.0%)
Income (by zip code)	
<$38,000	4348 (20.1%)
≥$38,000	17,245 (79.9%)
Education (by zip code)	
≥21% with no high school diploma	3874 (17.9%)
<21% with no high school diploma	17,719 (82.1%)
Metropolitan population type	16,991 (78.7%)
Distance from treatment center, miles (median)	8.7 (0–3691)
Center volume (cases/year)	
Top quartile	3.4–18.9
2nd quartile	1.84–3.3
3rd quartile	1.08–1.83
Bottom quartile	0.20–1.07
Clinical stage	
II	11,351 (52.6%)
III	10,242 (47.4%)
Histology	
Adenocarcinoma	11,249 (52.1%)
Squamous cell carcinoma	9068 (42.0%)
Unknown	1276 (5.9%)
Tumor location	
Cervical esophagus	724 (3.4%)
Upper third	1303 (6.0%)
Middle third	3545 (16.4%)
Lower third	12,239 (56.7%)
Unknown	3782 (17.5%)
Treatment type	
No treatment	2787 (12.9%)
Palliative therapy	5252 (24.3%)
Concurrent chemoradiation	8010 (37.1%)
Esophagectomy alone	1215 (5.6%)
Trimodality therapy	2159 (10.0%)
Unknown	2170 (10.1%)

### Treatment utilization and outcome

Among the treatment categories analyzed, definitive (concurrent) chemoradiation was the most common (37.1%) followed by palliative therapy (24.3%). In the definitive chemoradiation group, the mean elapsed time of radiation treatment was 42.0 ± 0.3 days and most (70.6%) received multiagent chemotherapy. Within the palliative therapy group, the majority received radiation alone (50.7%), followed by sequential chemoradiation (34.1%) and chemotherapy alone (15.2%). Ten percent received trimodality therapy. For the neoadjuvant portion, mean elapsed time of radiation treatment was 39.0 ± 0.8 days with 80.1% receiving multiagent chemotherapy. Trends in utilization of each over time are shown in Figure S1. Between 1998 and 2012, as compared to prior years, trimodality use steadily increased, while esophagectomy alone increased in early years, but declined significantly after 2009. Between age groups within the elderly cohort, there was heterogeneity in treatment utilization as well (Table 2). Notably, patients aged 80 and older were significantly more likely to receive palliative therapy or no treatment and less likely to receive trimodality therapy or surgery alone.

**Table 2:** Distribution of treatments by age group

Treatment type	Age at diagnosis (years)		*P* value
	70–79 (*n* = 14,580)	≥80 (*n* = 7013)	
No treatment	1386 (9.5%)	1401 (20.0%)	<0.001
Palliative therapy	3041 (20.9%)	2211 (31.5%)	
Concurrent chemoradiation	5528 (37.9%)	2482 (35.4%)	
Esophagectomy alone	912 (6.2%)	303 (4.3%)	
Trimodality therapy	2014 (13.8%)	145 (2.1%)	
Unknown	1699 (11.7%)	471 (6.7%)	

**Figure 2:**
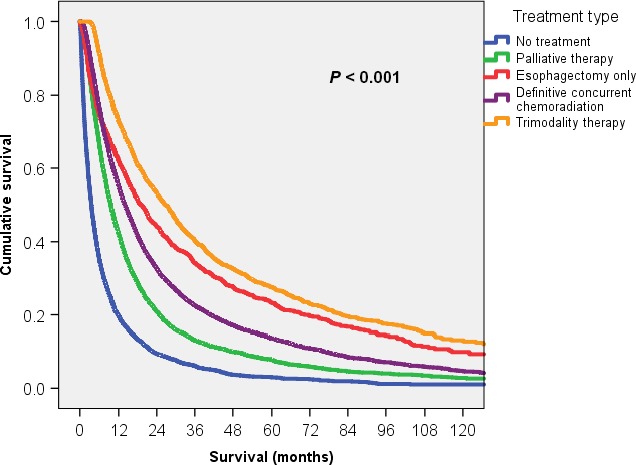
Kaplan–Meier overall survival for elderly patients with locally advanced esophageal cancer stratified by type of treatment.

**Figure 3:**
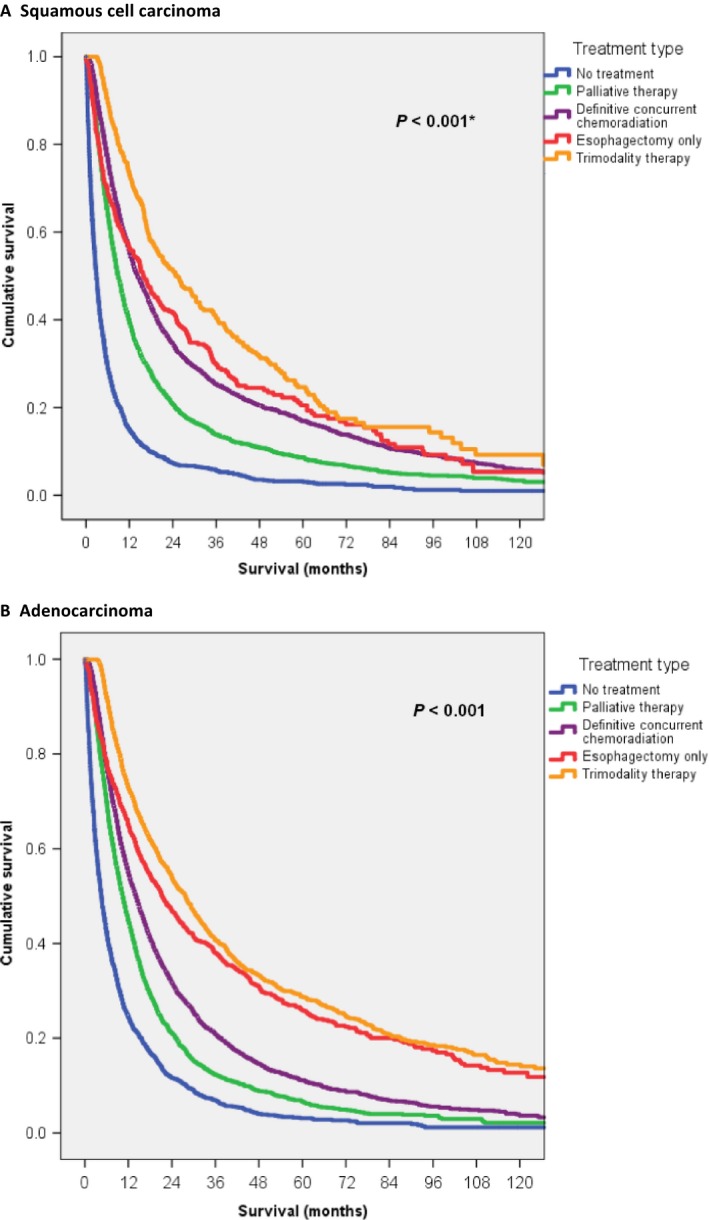
Kaplan–Meier overall survival for elderly patients with locally advanced squamous cell carcinoma (A) or adenocarcinoma (B) of the esophagus stratified by type of treatment. *P value for only the comparison between esophagectomy alone and definitive concurrent chemoradiation in squamous cell carcinoma patients was 0.44. All other comparisons were statistically significant.

Overall survival by treatment type is shown in Figure 2. Patients who underwent trimodality therapy had the most favorable outcome with a median survival of 26.8 months (95% CI: 24.9–28.7) followed by esophagectomy alone at 19.3 months (95% CI: 17.1–21.5) and chemoradiation at 14.0 months (95% CI: 13.5–14.5). Median survival for palliative therapy was 9.7 months (95% CI: 9.3–10.1). Those who did not undergo any tumor‐directed therapy had a median survival of 3.6 months (95% CI: 3.4–3.9). When separated by histology, survival trends by treatment type were generally comparable to the overall cohort with squamous cell carcinoma patients often trending toward worse survival than their adenocarcinoma counterparts (Fig. 3). As with the full cohort, the most favorable outcomes were seen with trimodality with median survival for squamous cell carcinoma of 24.8 months (95% CI: 19.7–30.0) and for adenocarcinoma of 27.7 months (95% CI: 25.5–30.0), and the least favorable outcomes were with no treatment with median survivals of 2.9 months (95% CI: 2.6–3.2) and 4.6 months (95% CI: 4.1–5.1), respectively. Interestingly, for squamous cell carcinoma only, median survival for esophagectomy alone (15.9 months, 95% CI: 12.3–19.5), though slightly higher than 14.3 months for concurrent chemoradiation, was not statistically significant (*P* = 0.44). Otherwise, median survivals were statistically different for the remaining treatment comparisons for squamous cell carcinoma and all treatment comparisons for adenocarcinoma (*P* < 0.001).

### Predictors of utilization – palliative therapy versus no treatment

On univariate analysis, the palliative therapy group was younger (age 70–79, 57.9% vs. 49.7%), more likely to be male (67.9% vs. 64.2%) and clinical stage II (52.0% vs. 45.9%), and lived closer to their treatment center (24.9 vs. 31.7 miles) (Table S1). On multivariate logistic regression, age ≥ 80 (OR 0.73), female gender (OR 0.81), Charlson‐Deyo comorbidity score ≥1 (OR 0.82–0.85), clinical stage III (OR 0.80), and receiving treatment at a high‐volume center (OR 0.83) were independently associated with a decreased likelihood of receiving palliative therapy (Table 3). Only higher levels of education (OR 1.25) or insurance status including private insurance (OR 1.55) and Medicare (OR 1.77) were independently associated with receiving palliative therapy. After propensity matching (1105 patient pairs), there was a significant improvement in median survival from 3.5 months (95% CI: 3.1–3.9) in the no treatment group to 9.9 months (95% CI: 9.1–10.6) in the palliative therapy group, *P* < 0.001 (Figs. S2andS3).

**Table 3:** Multivariate analyses of predictors of treatment utilization for definitive and palliative therapy in elderly patients with locally advanced esophageal cancer

Variable	Palliative			Definitive		
	Palliative therapy versus no treatment			Trimodality therapy versus concurrent chemoradiation		
	Odds ratio	95% CI	*P* value	Odds ratio	95% CI	*P* value
Age ≥80 (Ref: 70–79)	0.73	0.65–0.81	<0.001	0.15	0.12–0.18	<0.001
Female gender	0.81	0.72–0.91	<0.001	0.80	0.69–0.92	0.03
High‐volume center (Ref: low‐volume)	0.83	0.73–0.95	0.008	2.34	2.07–2.65	<0.001
Tumor location (Ref: lower third)						
Cervical	1.45	1.05–2.00	0.02	N/A		
Upper third	1.13	0.90–1.41	0.03	2.76	1.82–4.19	<0.001
Lower third	1.01	0.86–1.18	0.90	4.09	2.74–6.11	<0.001
Unknown	0.79	0.68–0.91	0.001	2.41	1.59–3.67	<0.001
Education level: <21% with no high school diploma (Ref: ≥21%)	1.25	1.08–1.45	0.003			
Insurance status (Ref: uninsured)						
Private	1.55	0.87–2.76	0.14			
Medicaid	1.52	0.73–3.14	0.26			
Medicare	1.77	1.01–3.09	0.045			
Other government	3.79	1.63–8.81	0.002			
Charlson‐Deyo score (Ref: 0)						
1	0.85	0.74–0.97	0.015			
≥2	0.82	0.67–0.99	0.04			
Clinical stage III (Ref: stage II)	0.80	0.72–0.90	<0.001			
Non‐Caucasian race (Ref: Caucasian)				0.63	0.48 to 0.81	<0.001
Income ≥$38,000 (Ref: <$38,000)				1.21	1.03–1.42	0.02
Adenocarcinoma histology (Ref: squamous)				2.10	1.80–2.45	<0.001

### Predictors of utilization – trimodality therapy versus definitive chemoradiation

On univariate analysis, the trimodality group was overwhelmingly younger (age 70–79, 93.3% vs. 68.8%) and more likely to be male (83.5% vs. 73.0%), Caucasian (95.9% vs. 90.0%), and treated at a high‐volume center (40.7% vs. 22.0%). Trimodality was more often associated with adenocarcinoma histology (72.6% vs. 51.3%) and tumors in the lower third of the esophagus (77.8% vs. 56.7%) (Table S2). Multivariate analysis confirmed that patients who were ≥80 years old (OR 0.15), female (OR 0.80), or non‐Caucasian (OR 0.63) were significantly less likely to receive trimodality, while those with adenocarcinoma histology (OR 2.10) or treated at a high‐volume center (OR 2.34) were more likely to receive trimodality (Table 3). After propensity matching (955 patient pairs), there was a significant improvement in median survival from 15.6 months (95% CI: 14.3–16.9) in the concurrent chemoradiation group to 27.6 months (95% CI: 24.7–30.4) among trimodality patients, *P* < 0.001 (Figs. S4andS5).

Because age was a strong predictor of not receiving trimodality therapy, we then explored perioperative morbidity and mortality between age groups. We found that mean length of hospital stay (12.8 vs. 14.3 days, *P* = 0.25) and 30‐day readmission rates (6.7% vs. 7.0%, *P* = 0.90) were not significantly different. On the other hand, 30‐day and 90‐day mortality were worse for patients ≥80 years old (10.4% vs. 5.5%, *P* = 0.03, and 23.7% vs. 14.2%, *P* = 0.006, respectively), though the number of these older patients is small (*n* = 145) relative to the entire cohort (Table S3).

### Predictors of utilization – esophagectomy alone versus definitive chemoradiation

While its use appears to have declined in recent years and trimodality is the more common surgical approach for definitive therapy in our cohort, esophagectomy alone is still utilized as definitive therapy in a portion of patients. Therefore, we also compared patients receiving esophagectomy alone to those receiving concurrent chemoradiation in the definitive setting. On univariate analysis, the esophagectomy group was younger (age 70–79, 75.1% vs. 69.0%), more likely to be male (77.4% vs. 72.3%), Caucasian (95.4% vs. 89.9%), and live farther from the treatment center (54.2 vs. 32.7 miles). As with trimodality, esophagectomy alone was also more often associated with adenocarcinoma histology (71.5% vs. 52.4%) and tumors in the lower third of the esophagus (81.6% vs. 68.8%) (Table S4). On multivariate logistic regression, age ≥80 (OR 0.79), clinical stage III (OR 0.33), and tumors in the upper and middle third of the esophagus (OR 0.31–0.69) were independently associated with a decreased likelihood, while those with a Charlson‐Deyo comorbidity score ≥1 (OR 1.31–1.47), adenocarcinoma histology (OR 1.33), and being treated at a nonacademic cancer center (OR 3.9) were more likely to receive esophagectomy alone (Table 4). After propensity matching (697 patient pairs), there was a significant improvement in median survival from 15.3 months (95% CI: 13.3–17.3) in the concurrent chemoradiation group to 19.8 months (95% CI: 16.5–23.2) among esophagectomy alone patients, *P* < 0.001 (Figs. S6andS7).

**Table 4:** Multivariate analysis of predictors of esophagectomy alone compared to definitive chemoradiation

Variable	Odds ratio	95% CI	*P* value
Age ≥80 (Ref: 70–79)	0.79	0.65–0.96	0.015
Clinical stage III (Ref: stage II)	0.33	0.27–0.40	<0.001
Charlson‐Deyo score (Ref: 0)			
1	1.31	1.07–1.61	0.01
≥2	1.47	1.07–2.04	0.02
Tumor location (Ref: lower third)			
Upper third	0.31	0.19–0.51	<0.01
Middle third	0.69	0.53–0.91	0.008
Adenocarcinoma histology (Ref: squamous)	1.33	1.07–1.67	0.012
Nonacademic cancer center (Ref: academic center)	3.9	3.2–4.6	<0.001

### Predictors of outcome

Risk factors for survival in the full elderly cohort were analyzed using a Cox proportional hazards model (Table 5). Increased overall mortality was associated with age ≥80 (HR 1.21), a Charlson‐Deyo comorbidity score ≥1 (HR 1.21–1.41), and clinical stage III (HR 1.31, reference: stage II). Decreased mortality hazard was associated with female gender (HR 0.94), higher income (HR 0.93), receiving care at a high‐volume center (HR 0.83), and adenocarcinoma histology (HR 0.94). Any tumor‐directed therapy resulted in an independent relative improvement in survival over no treatment with more substantial mortality hazard reduction being achieved for each therapy type: palliative therapy (HR 0.49), definitive chemoradiation (HR 0.36), esophagectomy (HR 0.31), and trimodality (HR 0.25), respectively.

**Table 5:** Hazard ratios for death for elderly patients with locally advanced esophageal cancer

Variable	Hazard ratio	95% CI	*P* value
Age ≥80 (Ref: 70–79)	1.21	1.15–1.27	<0.001
Female Gender	0.94	0.90–0.98	0.009
Income ≥$38,000 (Ref: <$38,000)	0.93	0.88–0.98	0.006
Charlson‐Deyo score (Ref: 0)			
1	1.21	1.15–1.27	<0.001
≥2	1.42	1.31–1.53	<0.001
High‐volume center (Ref: low‐volume)	0.83	0.79–0.87	<0.001
Clinical stage III (Ref: stage II)	1.31	1.26–1.37	<0.001
Adenocarcinoma histology (Ref: squamous)	0.94	0.90–0.98	0.03
Treatment type (Ref: no treatment)			
Palliative therapy	0.49	0.46–0.53	<0.001
Concurrent chemoradiation	0.36	0.34–0.39	<0.001
Esophagectomy alone	0.31	0.28–0.34	<0.001
Trimodality therapy	0.25	0.23–0.27	<0.001

Since adenocarcinoma histology was associated with improved survival and was a very strong predictor of receiving trimodality therapy, we then explored outcomes after definitive treatment based on histology. In the unmatched cohort, median overall survival with trimodality therapy was 27.7 months (95% CI: 25.5–30.0) in patients with adenocarcinoma and 24.8 months (95% CI: 19.7–30.0) in patients with squamous cell carcinoma; however, this difference was not statistically significant (*P* = 0.14, S8A). For those treated with concurrent chemoradiation, median survivals of patients with adenocarcinoma and squamous cell histology, while significantly different, were days apart at 14.1 and 14.3 months, respectively (*P* < 0.001) (Table S5, Figure S8B).

## Discussion

Among a modern cohort of elderly patients with locally advanced esophageal cancer, our results demonstrate significant variability in treatment utilization and associated outcomes based on demographic and tumor characteristics. Treatment decisions were significantly influenced by age, sex, and treatment center patient volume. Race, comorbidities, education, income, and tumor location and histology also impacted utilization in specific scenarios. Tumor‐directed therapy resulted in improved survival over no therapy with incremental improvement as the aggressiveness of treatment increased from palliative therapy to definitive chemoradiation to surgical management of disease.

Within the elderly cohort, age was one of the strongest predictors for treatment utilization and survival. Patients ≥80 years old were more likely to receive no treatment in the nondefinitive setting and much less likely to receive trimodality therapy and esophagectomy alone when treated definitively. Underlying these differences is likely a combination of patient‐related factors, physician bias, and clinical realities. Numerous studies across various malignancies have shown that advanced age is associated with decreased referral to specialists, increased delivery of suboptimal therapy, and increased patient refusal of therapy 16, 17, 18, 19. Objective clinical reasons supporting a more cautious approach to older individuals include decreased stem‐cell reserves and the presence of comorbidities impacting drug absorption and/or metabolism 20, 21. Additionally, esophagectomy has been associated with higher rates of perioperative mortality in the elderly 5. Indeed, we observed a higher percentage of 30‐ and 90‐day mortality in the ≥80 age group, though the absolute number of deaths is smaller than the 70–79‐year‐old group and the rates in the ≥80 group may be overestimated due to the relatively small number of patients in that age group (~6% of the trimodality cohort). Nevertheless, in appropriately selected individuals, morbidity and mortality from esophagectomy is comparable to younger patients in other studies, even for octogenarians 22, 23. As it relates to the impact on survival, our data support the use of more aggressive therapy, including surgery, in the elderly patient with locally advanced esophageal cancer. While patient selection can impact these findings, we do see consistent improvement in outcomes with more aggressive therapy in matched cohorts as well.

Gender was also a predictor of treatment utilization and survival with women appearing to be treated less aggressively. The presence and causes of gender disparities in cancer treatment are not well defined, in part because many malignancies are gender‐specific. However, a SEER‐based analysis on colorectal cancer patients found that women, particularly octogenarians, underwent less aggressive therapy than men 24. Despite this, women in this study and in our cohort had improved survival 24. Factors underlying this contradiction are not clear, especially since women in our cohort were older (mean age 78.2 vs. 77, *P* < 0.001). While a difference in death from intercurrent disease is likely a contributor, gender differences in disease progression and treatment response are an underexplored possibility. Our results argue that care should be taken not to avoid aggressive therapy in elderly females with locally advanced esophageal cancer as their survival is comparable if not improved from their male counterparts.

In our elderly cohort, a significant percentage (37.2%) is not given definitive therapy for locally advanced disease. Of these patients, we see a near threefold improvement in median survival with palliative treatment compared to no therapy. The benefit of a particular palliative strategy is difficult to ascertain due to the heterogeneity of treatment approaches, but a large majority (~85%) received radiation therapy and nearly half received chemotherapy. Additionally, the improved survival with palliative therapy can be impacted by patient selection factors not well appreciated in the NCDB (e.g., performance status) or by including patients not expressly coded as palliative and thus potentially being treated more aggressively in a definitive‐type manner. Nevertheless, any nonconcurrent chemoradiation strategy is in practice a palliative therapy for locally advanced disease. And while the true benefit of palliative therapy is unclear, our data suggest that tumor‐directed therapy, even potentially with modest treatment such as radiation alone can have a significant impact on survival. Importantly, the impact of these therapies on quality of life is not well characterized and should continue to be an important variable in deciding whether or not to offer palliative treatment.

In the definitive setting, there has been a shift away from upfront esophagectomy to trimodality with neoadjuvant chemoradiation, particularly after the CROSS trial demonstrated improved outcomes over surgery alone 7. While patients over 75 years old were excluded in this study, this trend is nonetheless mirrored in our elderly cohort where we see increased utilization of trimodality from 2006 (6.7% of treatments) compared to 2012 (13.6%). In this regard, our cohort is more aligned with current practice than previous population‐based analyses of elderly patients where only 7% received trimodality 15, 16. Of note, the CROSS trial demonstrated a wider differential in median survival between trimodality and esophagectomy alone compared to our cohort (49 months vs. 24 months in the CROSS trial compared to 27 months vs. 19 months). This is most likely primarily due to the (intended) advanced age of our cohort with a 17‐year increase in median age (60 vs. 77) and the increased likelihood of competing risks as a result, though trimodality still appears to be associated with an improved survival in these elderly patients. Nevertheless, a small percentage of patients (5.6%) were treated with esophagectomy alone, and this may be due to a preference for surgical management of locally advanced disease as an alternative to chemoradiation in patients who are not felt to be appropriate candidates for trimodality therapy. Among our entire cohort, patients treated with esophagectomy alone did exhibit improved overall survival over chemoradiation, though this benefit may be more apparent in those with adenocarcinoma specifically.

Among patients receiving trimodality, we see a strong histologic bias toward adenocarcinomas. While adenocarcinoma histology was also associated with improved survival, there was no difference in survival between histologies within the trimodality group. The improved hazards ratio for adenocarcinoma, therefore, is likely the results of increased (threefold) utilization of a more effective treatment. Nevertheless, factors underlying this bias toward adenocarcinoma for trimodality are unclear. One possibility is the differences in tumor location and associated differences in surgical approaches and complexities, and this may explain the bias toward adenocarcinoma in patients receiving esophagectomy alone as well. Squamous cell carcinomas are more often in the mid and proximal esophagus where surgery is more extensive or significantly morbid 25. Other possibilities include the epidemiologic differences between the two histologies and the growing evidence that in squamous cell carcinoma, definitive chemoradiation can result is comparable survival to treatments involving surgery 26, 27, 28. Indeed, when we evaluated overall survival in the full cohort by histology and examined squamous cell carcinoma specifically, esophagectomy alone appears to offer no significant survival advantage over definitive chemoradiation.

Limitations of our study include the inability to reliably account for important factors such as comorbidities and performance status. This limits our ability to account for important patient selection criteria in treatment utilization, and can have a subsequent impact on survival analysis, though we attempted to mitigate this effect through propensity matching. Additionally, quality of life outcomes are an important determinant in this population, but are unable to be garnered from the NCDB. Finally, in the elderly when there is more likelihood of competing comorbidities, the inability to ascertain disease or progression‐free survival is a further limitation when evaluating the benefit of therapy. This effect is somewhat offset by the aggressiveness of locally advanced esophageal cancer (median survival of 2.2 years with trimodality) relative to the average life expectancy in this age group (4–17 years) 29.

In conclusion, our analysis of the NCDB demonstrates that elderly patients with clinical stage II and III esophageal cancer exhibited a survival benefit from any tumor‐directed therapy, including palliative treatment. The use of trimodality confers the largest survival benefit and its use has increased over time. Despite these improved outcomes with treatment, there are still numerous factors including age, gender, and histology as well as treatment at high‐ or low‐volume centers that significantly impact treatment utilization and care should be taken to avoid bias in determining the most appropriate therapy for the elderly patient with locally advanced esophageal cancer.

## Conflict of Interest

There are no conflicts of interest directly related to the contents of the manuscript from any author.

## Supporting information


**Figure S1.** Relative utilization of each treatment over time. For each treatment group, the percentage of patients in that group is plotted by year of diagnosis as a function of all patients receiving that treatment. Percentages generally increase over time as patient numbers increase, with the relative use of trimodality outpacing other treatment.**Figure S2.** Kaplan–Meier overall survival from propensity‐matched elderly patients with locally advanced esophageal cancer receiving palliative therapy or no treatment.**Figure S3.** Dot‐plot (A) and propensity histograms (B) for propensity‐matched elderly patients with locally advanced esophageal cancer receiving palliative therapy or no treatment.**Figure S4.** Kaplan–Meier overall survival from propensity‐matched elderly patients with locally advanced esophageal cancer receiving concurrent chemoradiation or trimodality therapy.**Figure S5.** Dot‐plot (A) and propensity histograms (B) for propensity‐matched elderly patients with locally advanced esophageal cancer receiving concurrent chemoradiation or trimodality therapy.**Figure S6.** Kaplan–Meier overall survival from propensity‐matched elderly patients with locally advanced esophageal cancer receiving concurrent chemoradiation or esophagectomy alone.**Figure S7.** Dot‐plot (A) and propensity histograms (B) for propensity‐matched elderly patients with locally advanced esophageal cancer concurrent chemoradiation or esophagectomy alone.**Figure S8.** Kaplan–Meier overall survival of elderly patients with locally advanced esophageal cancer receiving concurrent chemoradiation or trimodality by histology.

**Table S1.** Univariate analysis of predictors of palliative therapy. Within each variable, patients with unknown values were excluded and the number of remaining patients (*n*) was noted within each variable and treatment group when applicable.**Table S2.** Univariate analysis of predictors of trimodality therapy. Within each variable, patients with unknown values were excluded and the number of remaining patients (*n*) was noted within each variable and treatment group when applicable.**Table S3.** Perioperative outcomes in patients undergoing trimodality therapy by age group.**Table S4.** Univariate analysis of predictors of esophagectomy alone. Within each variable, patients with unknown values were excluded and the number of remaining patients (*n*) was noted within each variable and treatment group when applicable.**Table S5.** Survival after definitive therapy based on tumor histology.
