# Supplementary material for: Treatment utilization and outcomes in elderly patients with locally advanced esophageal carcinoma: a review of the National Cancer Database
Source: Cancer Med. 2017 Nov 15;6(12):2886–96. doi: 10.1002/cam4.1250 (PMC5727236; doi:10.1002/cam4.1250)

Supplemental Figure 1. Relative Utilization of Each Treatment Over Time. For each treatment group, the percentage of patients in that group are plotted by year of diagnosis as a function of all patients receiving that treatment. Percentages generally increase over time as patient numbers increase, with the relative use of trimodality outpacing other treatment types.

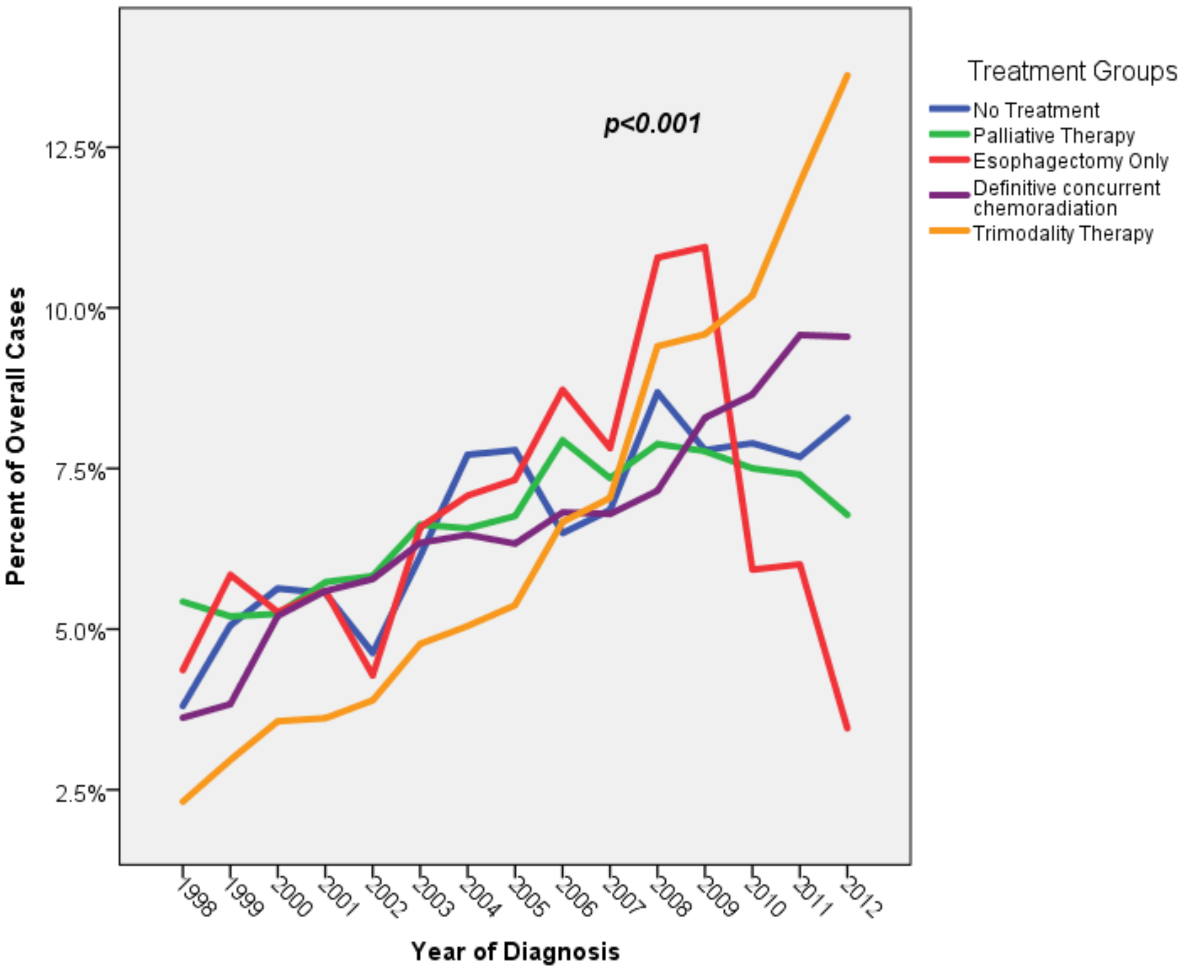

Supplemental Figure 2. Kaplan-Meier overall survival from Propensity Matched Elderly Patients with Locally Advanced Esophageal Cancer Receiving Palliative Therapy or No Treatment.

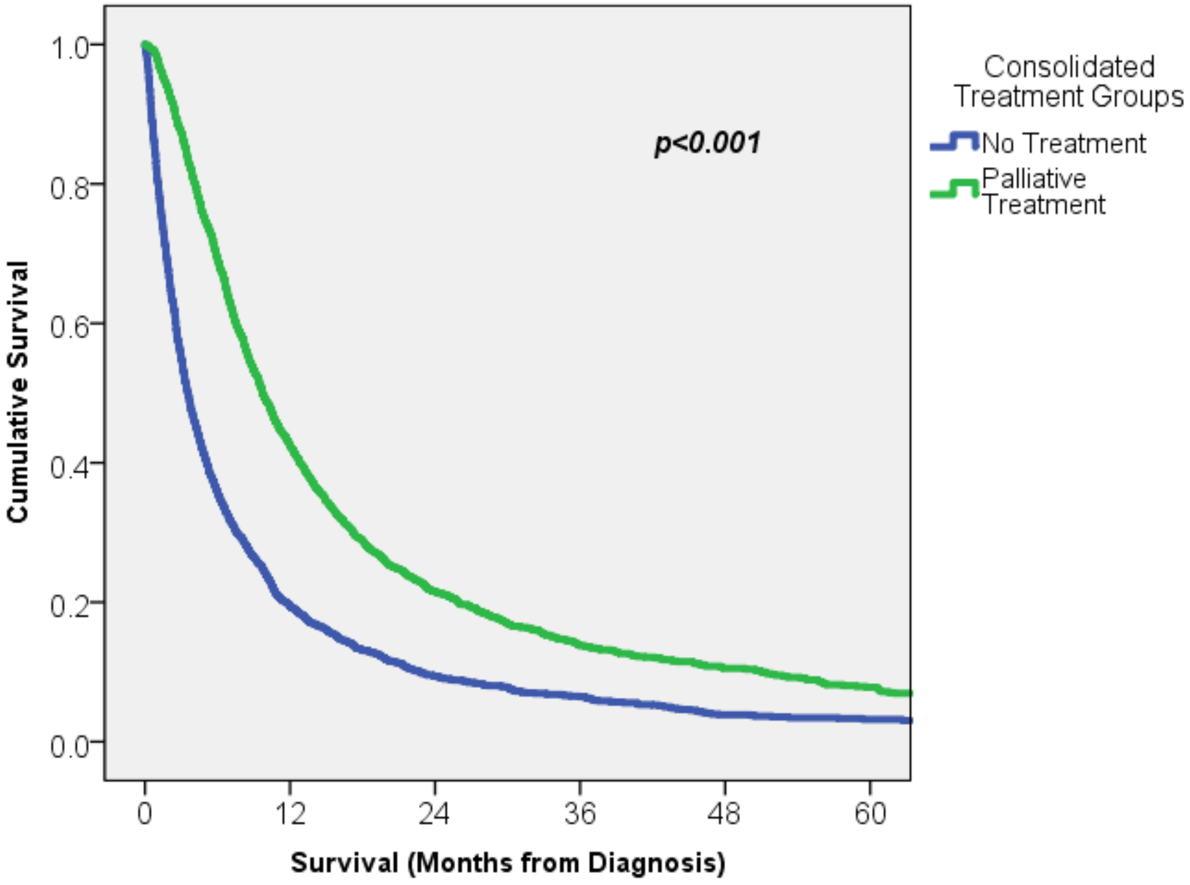

Supplemental Figure 3. Dot-Plot (A) and Propensity Histograms (B) for Propensity Matched Elderly Patients with Locally Advanced Esophageal Cancer Receiving Palliative Therapy or No Treatment.

A.

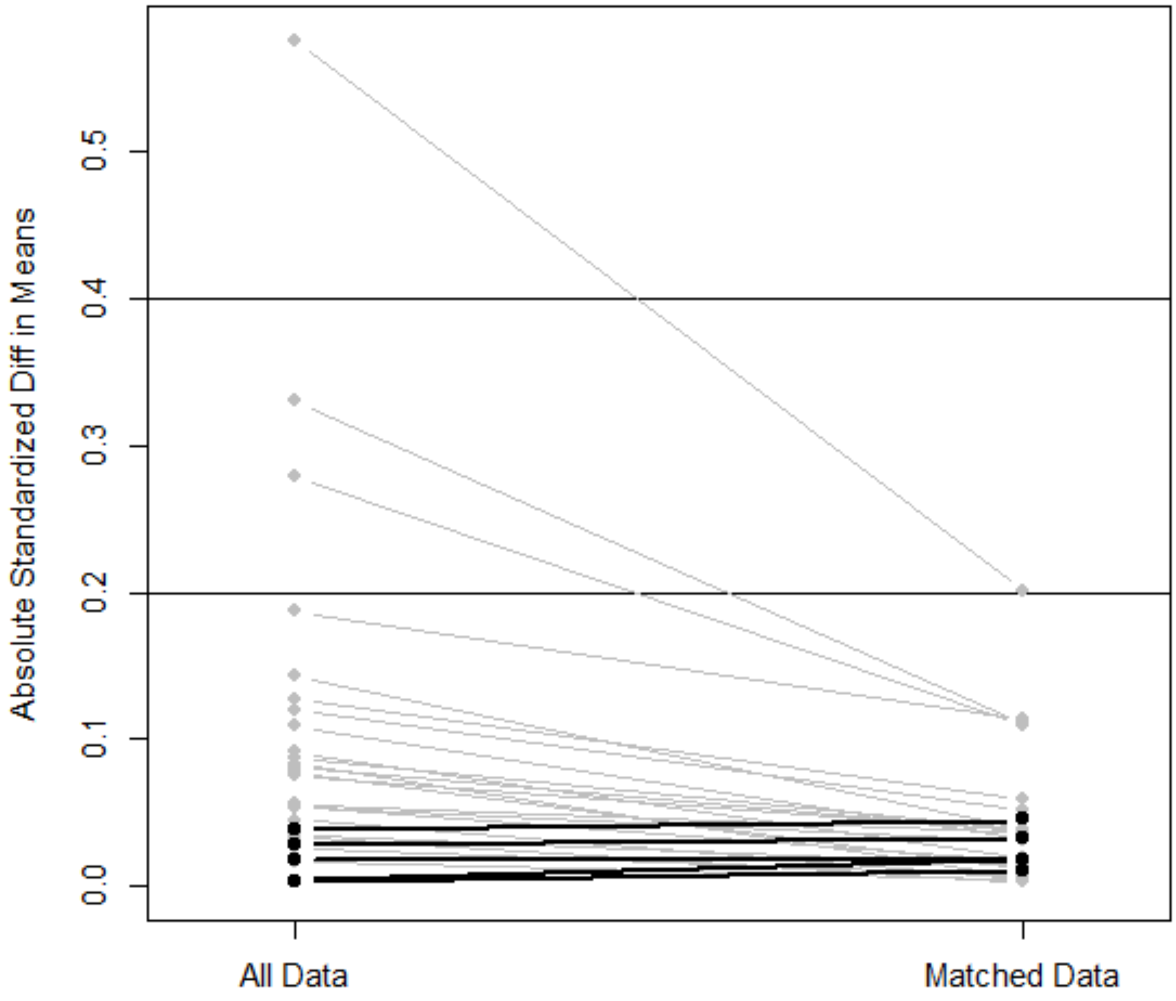

B.

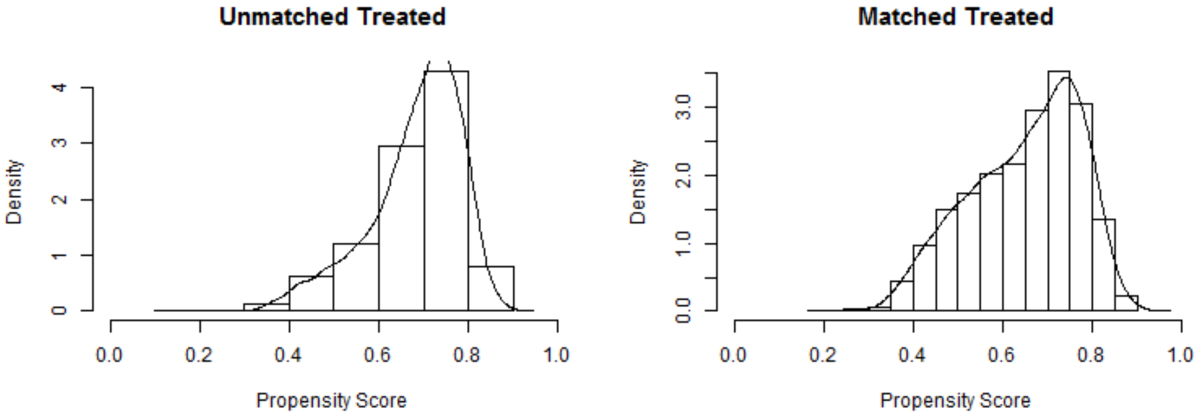

B (continued).

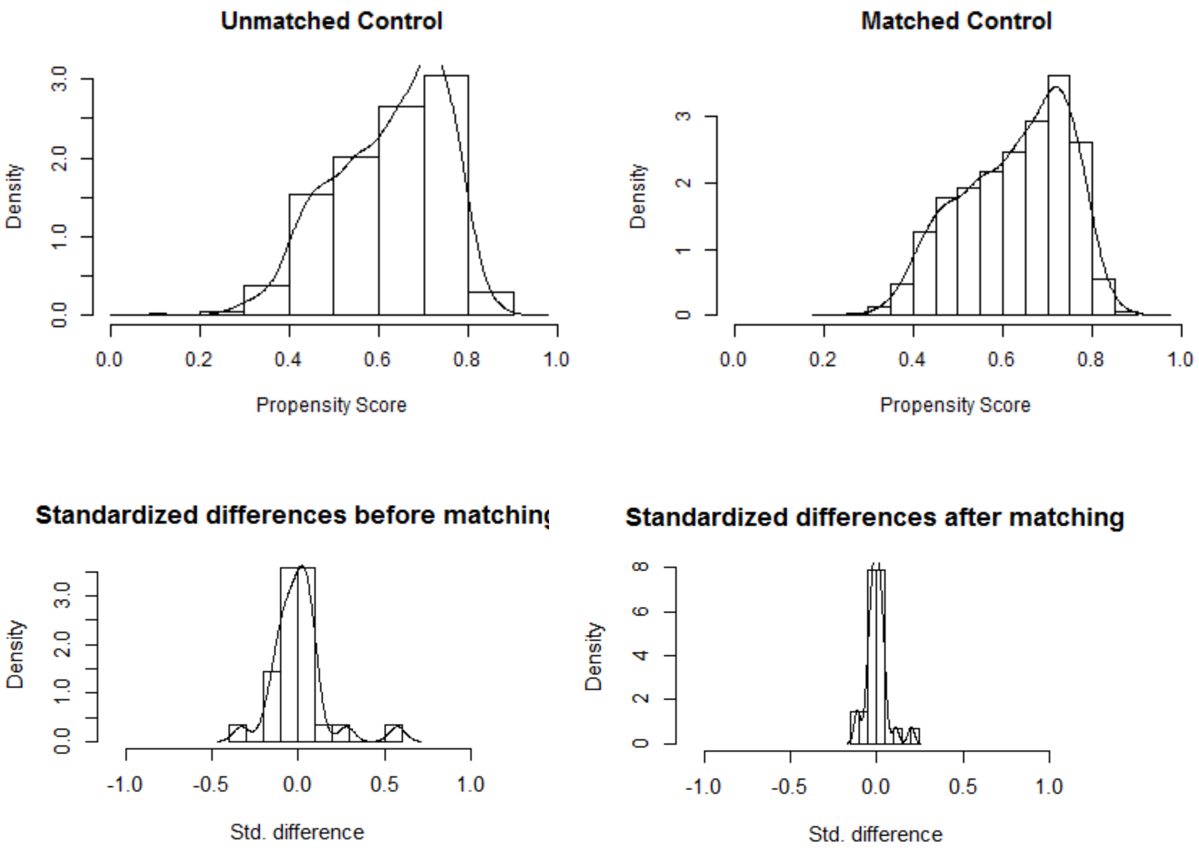
Unmatched Control
Density
3.0
2.0
1.0
0.0
0.0
0.2
0.4
0.6
0.8
1.0
Propensity Score
Matched Control
Density
3
2
1
0
0.0
0.2
0.4
0.6
0.8
1.0
Propensity Score
Standardized differences before matching
Density
3.0
2.0
1.0
0.0
-1.0
-0.5
0.0
0.5
1.0
Std. difference
Standardized differences after matching
Density
8
6
4
2
0
-1.0
-0.5
0.0
0.5
1.0
Std. difference

Supplemental Figure 4. Kaplan-Meier overall survival from Propensity Matched Elderly Patients with Locally Advanced Esophageal Cancer Receiving Concurrent Chemoradiation or Trimodality Therapy.

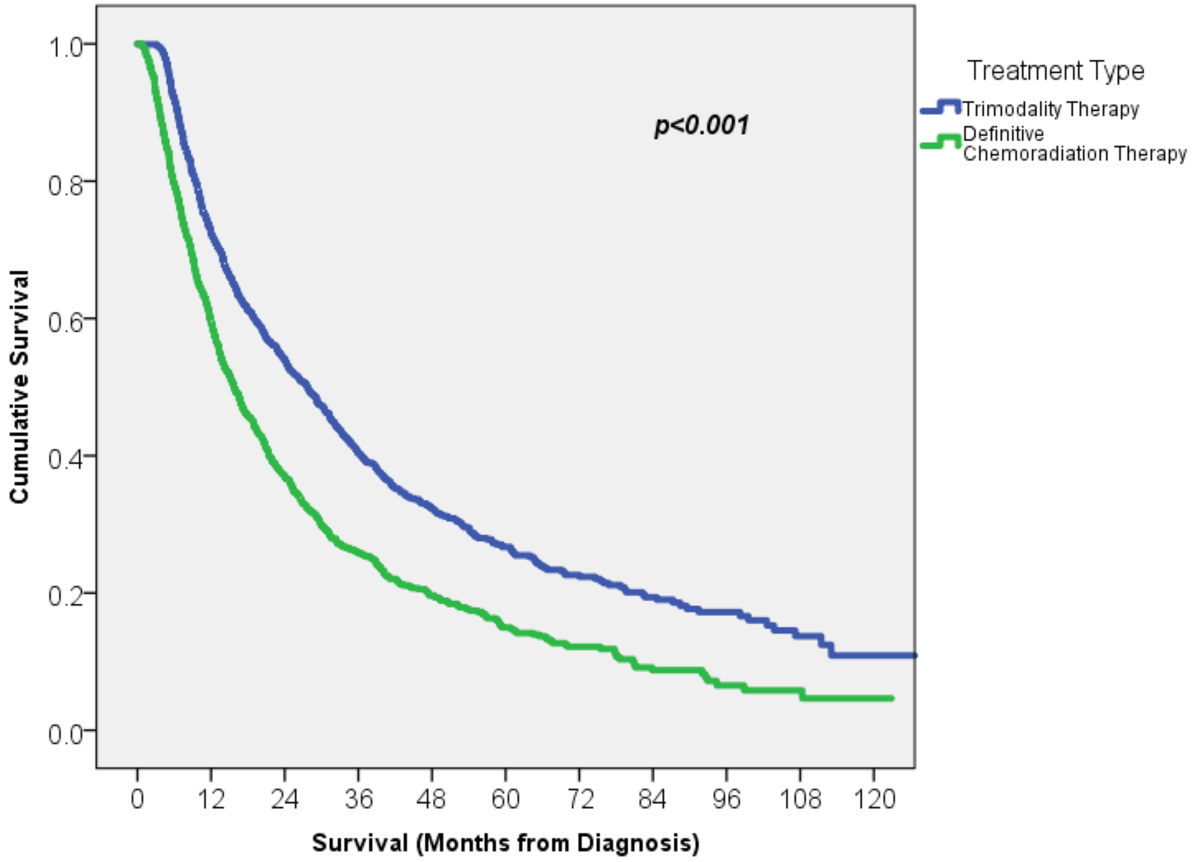

Supplemental Figure 5. Dot-Plot (A) and Propensity Histograms (B) for Propensity Matched Elderly Patients with Locally Advanced Esophageal Cancer Receiving Concurrent Chemoradiation or Trimodality Therapy.

A.

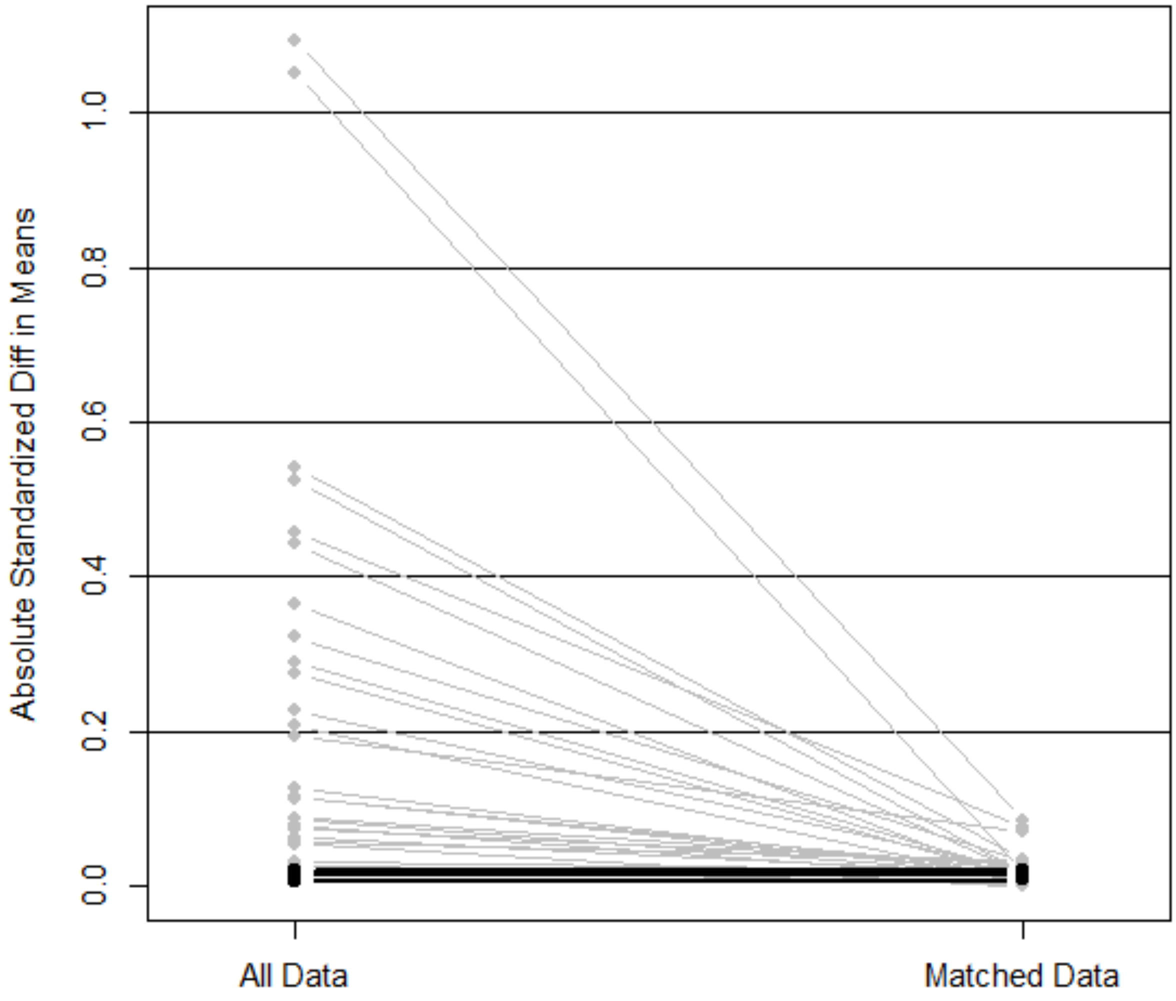

B.

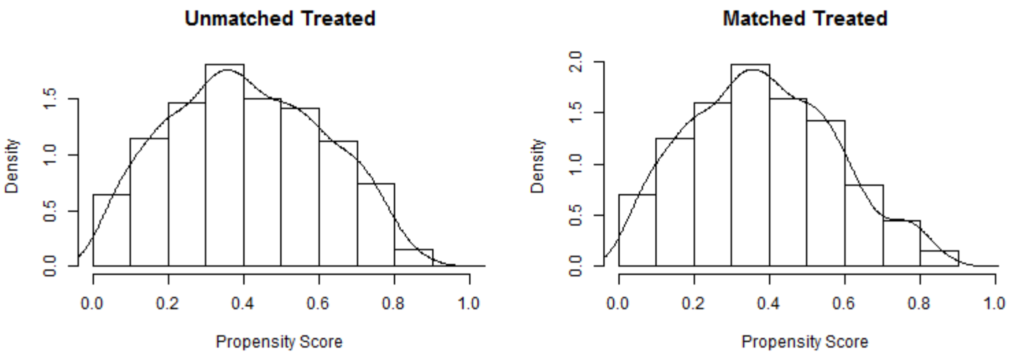

B (continued).

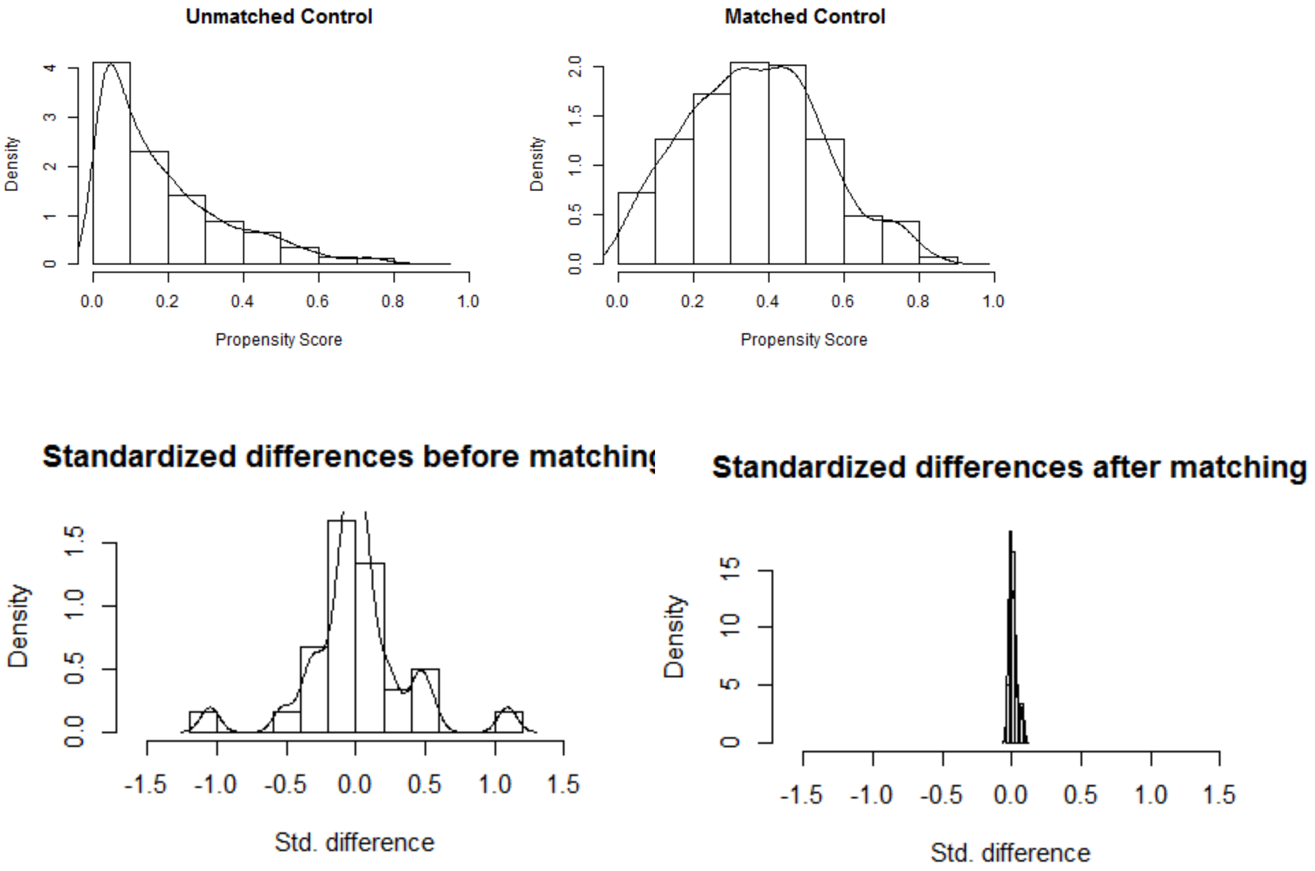

Unmatched Control
Density
0
1
2
3
4
0.0
0.2
0.4
0.6
0.8
1.0
Propensity Score
Matched Control
Density
0.0
0.5
1.0
1.5
2.0
0.0
0.2
0.4
0.6
0.8
1.0
Propensity Score
Standardized differences before matchin
Density
0.0
0.5
1.0
1.5
-1.5
-1.0
-0.5
0.0
0.5
1.0
1.5
Std. difference
Standardized differences after matching
Density
0
5
10
15
-1.5
-1.0
-0.5
0.0
0.5
1.0
1.5
Std. difference

Supplemental Figure 6. Kaplan-Meier overall survival from Propensity Matched Elderly Patients with Locally Advanced Esophageal Cancer Receiving Concurrent Chemoradiation or Esophagectomy Alone.

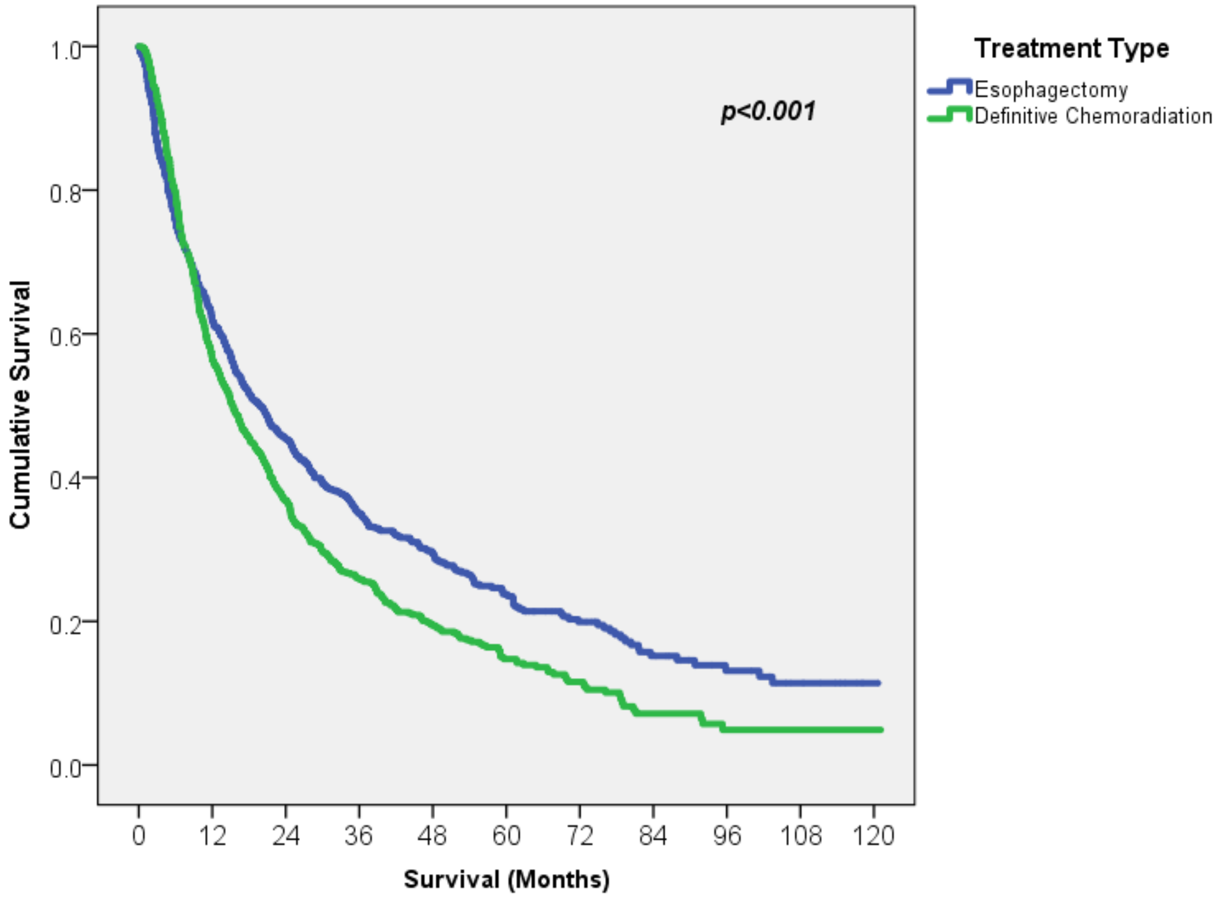

Supplemental Figure 7. Dot-Plot (A) and Propensity Histograms (B) for Propensity Matched Elderly Patients with Locally Advanced Esophageal Cancer Concurrent Chemoradiation or Esophagectomy Alone.

A.

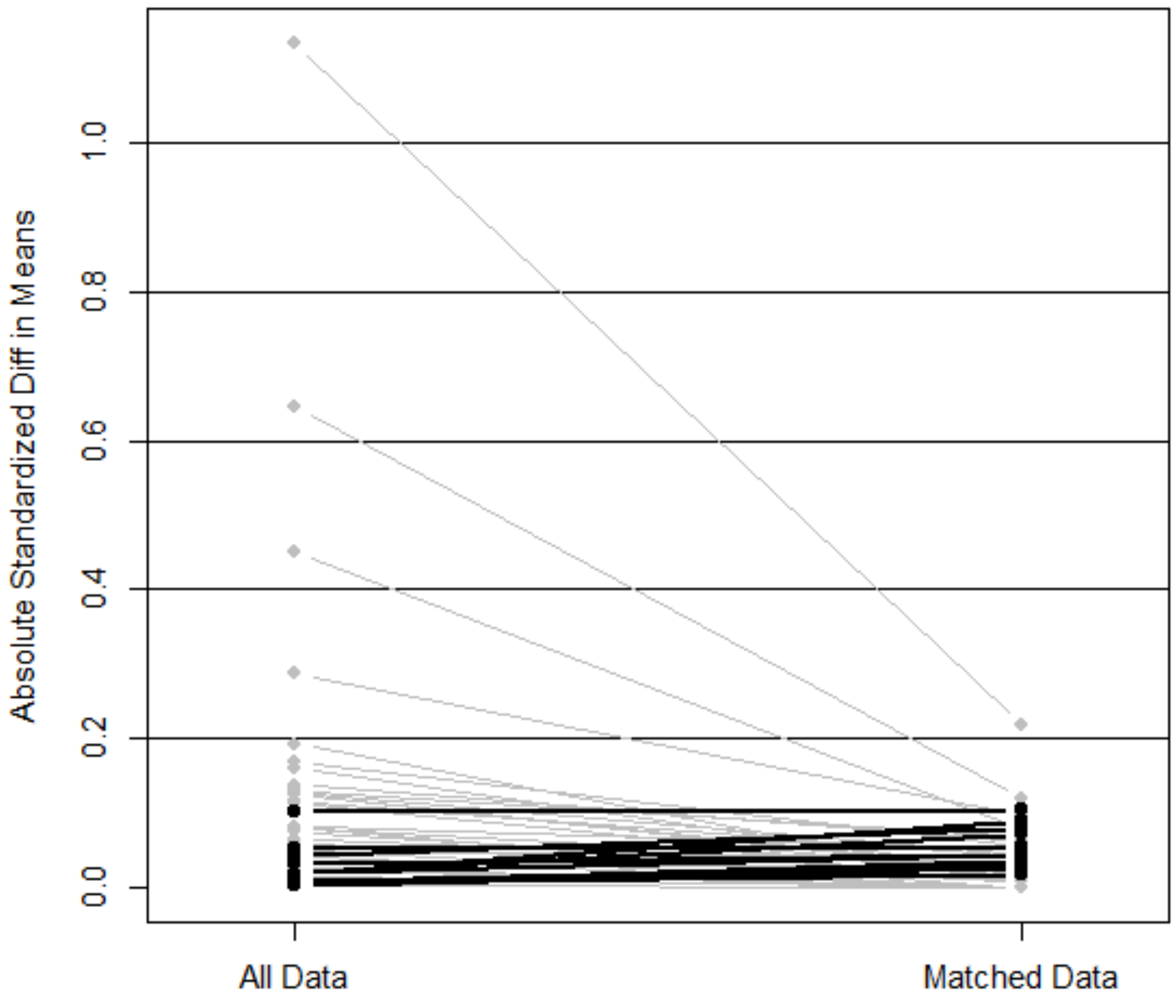

B.

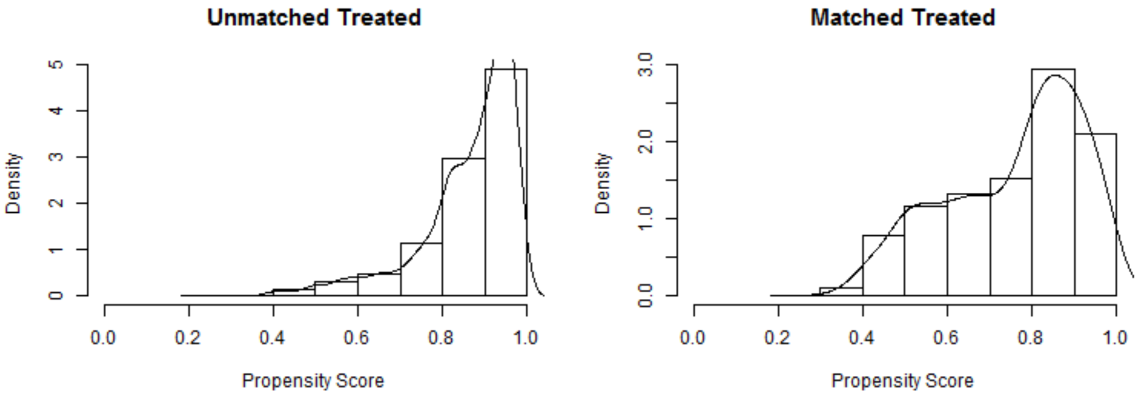

B (continued).

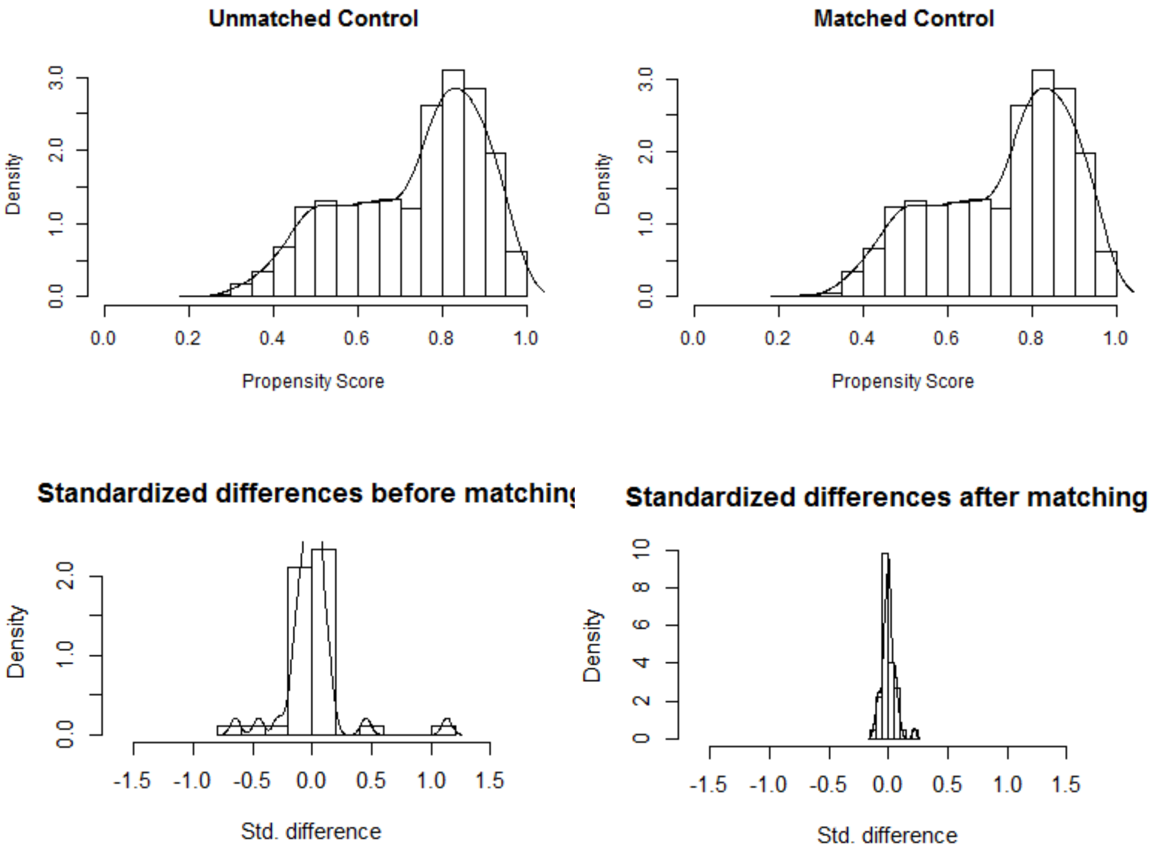
Unmatched Control
Density
3.0
2.0
1.0
0.0
0.0
0.2
0.4
0.6
0.8
1.0
Propensity Score
Matched Control
Density
3.0
2.0
1.0
0.0
0.0
0.2
0.4
0.6
0.8
1.0
Propensity Score
Standardized differences before matching
Density
2.0
1.0
0.0
-1.5
-1.0
-0.5
0.0
0.5
1.0
1.5
Std. difference
Standardized differences after matching
Density
10
8
6
4
2
0
-1.5
-1.0
-0.5
0.0
0.5
1.0
1.5
Std. difference

Supplemental Figure 8. Kaplan-Meier overall survival of Elderly Patients with Locally Advanced Esophageal Cancer Receiving Concurrent Chemoradiation or Trimodality by Histology.

A. Trimodality Therapy

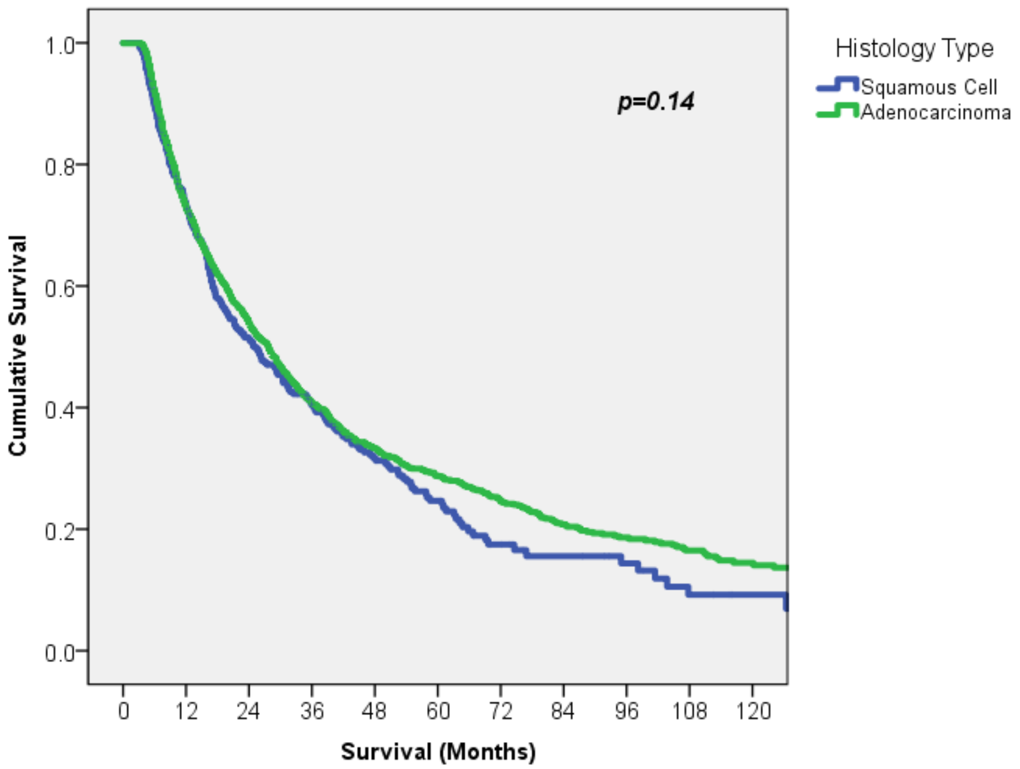

B. Definitive Chemoradiation

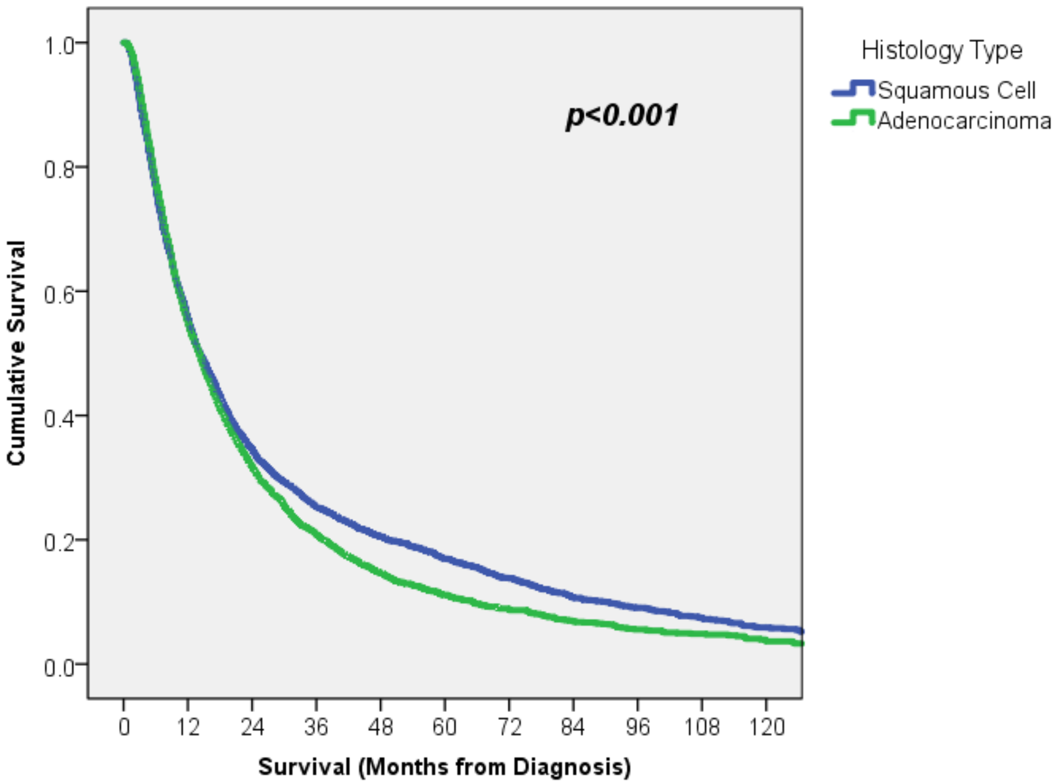

Supplement: Supplementary file 1 — Figure S1. Relative utilization of each treatment over time. For each treatment group, the percentage of patients in that group is plotted by year of diagnosis as a function of all patients receiving that treatment. Percentages generally increase over time as patient numbers increase, with the relative use of trimodality outpacing other treatment.Figure S2. Kaplan–Meier overall survival from propensity‐matched elderly patients with locally advanced esophageal cancer receiving palliative therapy or no treatment.Figure S3. Dot‐plot (A) and propensity histograms (B) for propensity‐matched elderly patients with locally advanced esophageal cancer receiving palliative therapy or no treatment.Figure S4. Kaplan–Meier overall survival from propensity‐matched elderly patients with locally advanced esophageal cancer receiving concurrent chemoradiation or trimodality therapy.Figure S5. Dot‐plot (A) and propensity histograms (B) for propensity‐matched elderly patients with locally advanced esophageal cancer receiving concurrent chemoradiation or trimodality therapy.Figure S6. Kaplan–Meier overall survival from propensity‐matched elderly patients with locally advanced esophageal cancer receiving concurrent chemoradiation or esophagectomy alone.Figure S7. Dot‐plot (A) and propensity histograms (B) for propensity‐matched elderly patients with locally advanced esophageal cancer concurrent chemoradiation or esophagectomy alone.Figure S8. Kaplan–Meier overall survival of elderly patients with locally advanced esophageal cancer receiving concurrent chemoradiation or trimodality by histology. [file CAM4-6-2886-s001.pdf]
