# Supplementary material for: Treatment utilization and outcomes in elderly patients with locally advanced esophageal carcinoma: a review of the National Cancer Database
Source: Cancer Med. 2017 Nov 15;6(12):2886–96. doi: 10.1002/cam4.1250 (PMC5727236; doi:10.1002/cam4.1250)
Supplement: Supplementary file 2 — Table S1. Univariate analysis of predictors of palliative therapy. Within each variable, patients with unknown values were excluded and the number of remaining patients (n) was noted within each variable and treatment group when applicable.Table S2. Univariate analysis of predictors of trimodality therapy. Within each variable, patients with unknown values were excluded and the number of remaining patients (n) was noted within each variable and treatment group when applicable.Table S3. Perioperative outcomes in patients undergoing trimodality therapy by age group.Table S4. Univariate analysis of predictors of esophagectomy alone. Within each variable, patients with unknown values were excluded and the number of remaining patients (n) was noted within each variable and treatment group when applicable.Table S5. Survival after definitive therapy based on tumor histology. [file CAM4-6-2886-s002.docx]

Supplemental Table 1. Univariate Analysis of Predictors of Palliative Therapy. Within each variable, patients with unknown values were excluded and the number of remaining patients (n) was noted within each variable and treatment group when applicable.

| **Variable** | **No Treatment**  **(n = 2,787)** | **Palliative Therapy**  **(n=5,252)** | **P Value** |
| --- | --- | --- | --- |
| Age Group  70-79 years  ≥80 years | 1,386 (49.7%)  1,401 (50.3%) | 3,041 (57.9%)  2,211 (42.1%) | <0.001 |
| Sex  Male  Female | 1,788 (64.2%)  999 (35.8%) | 3,565 (67.9%)  1,687 (32.1%) | 0.001 |
| Race  Caucasian  Non-Caucasian | 2,409 (86.4%)  378 (13.6%) | 4,576 (87.1%)  676 (12.9%) | 0.39 |
| Insurance Status  Uninsured  Private  Medicaid  Medicare  Other Government | *n = 2,718*  30 (1.1%)  323 (11.9%)  42 (1.5%)  2,309 (85.0%)  14 (0.5%) | *n = 5,103*  42 (0.8%)  573 (11.2%)  70 (1.4%)  4,349 (85.2%)  69 (1.4%) | 0.006 |
| Income (by zip code)  <$38,000  ≥$38,000 | 538 (19.3%)  2,249 (80.7%) | 1,014 (19.3%)  4,238 (80.7%) | 0.99 |
| Education Level (by zip code)  ≥21% with no high school diploma  <21% with no high school diploma | 524 (18.8%)  2,263 (81.2%) | 880 (16.8%)  4,372 (83.2%) | 0.02 |
| Population Type  Metropolitan  Urban  Rural | *n = 2,647*  2,237 (84.5%)  351 (13.3%)  59 (2.2%) | *n = 5,014*  4,226 (84.3%)  705 (14.1%)  83 (1.7%) | 0.14 |
| Greatest circle distance from treatment center (miles) | 31.7 ± 147.9 | 24.9 ± 112.1 | 0.04 |
| Treatment Center Volume  High (≥3.4 cases/yr)  Low (<3.4 cases/yr) | 617 (22.1%)  2,170 (77.9%) | 1034 (19.7%)  4,218 (80.3%) | 0.01 |
| Charlson-Deyo Score  0  1  ≥2 | *n = 2,099*  1,409 (67.1%)  494 (23.5%)  196 (9.3%) | *n = 3,812*  2,684 (70.4%)  815 (21.4%)  313 (8.2%) | 0.03 |
| Clinical Stage  II  III | 1,279 (45.9%)  1,508 (54.1%) | 2,733 (52.0%)  2,519 (48.0%) | <0.001 |
| T stage (clinical)  T1  T2  T3  T4 | *n = 2,573*  187 (7.3%)  619 (24.1%)  1,079 (41.9%)  688 (26.7%) | *n = 4,983*  204 (4.1%)  1,323 (26.6%)  2,645 (53.1%)  811 (16.3%) | <0.001 |
| N stage (clinical)  N0  N1  N2  N3 | *n = 2,547*  1,163 (45.7%)  1,305 (51.2%)  59 (2.3%)  20 (0.8%) | *n = 4,953*  2,377 (48.0%)  2,400 (48.5%)  142 (2.9%)  34 (0.7%) | 0.08 |
| Tumor Location  Cervical  Upper Third  Middle Third  Lower Third | *n = 2,131*  91 (4.3%)  198 (9.3%)  490 (23.0%)  1,352 (63.4%) | *n = 4,234*  231 (5.5%)  390 (9.2%)  969 (22.9%)  2,644 (62.4%) | <0.001 |
| Histology  Adenocarcinoma  Squamous Cell Carcinoma | *n = 2,654*  1,364 (51.4%)  1,290 (48.6%) | *n = 4,972*  2,585 (52.0%)  2,387 (48.0%) | 0.62 |

Supplemental Table 2. Univariate Analysis of Predictors of Trimodality Therapy. Within each variable, patients with unknown values were excluded and the number of remaining patients (n) was noted within each variable and treatment group when applicable.

| **Variable** | **Concurrent Chemoradiation**  **(n = 7,698)** | **Trimodality Therapy**  **(n=2,156)** | **P Value** |
| --- | --- | --- | --- |
| Age Group  70-79 years  ≥80 years | 5,296 (68.8%)  2,402 (31.2%) | 2,011 (93.3%)  145 (6.7%) | <0.001 |
| Sex  Male  Female | 5,618 (73.0%)  2,080 (27.0%) | 1,801 (83.5%)  355 (16.5%) | <0.001 |
| Race  Caucasian  Non-Caucasian | 6,925 (90.0%)  773 (10.0%) | 2,067 (95.9%)  89 (4.1%) | <0.001 |
| Insurance Status  Uninsured  Private  Medicaid  Medicare  Other Government | *n = 7,384*  42 (0.6%)  789 (10.7%)  90 (1.2%)  6,353 (86.0%)  110 (1.5%) | *n = 2,096*  8 (0.4%)  267 (12.7%)  14 (0.7%)  1,791 (85.4%)  16 (0.8%) | 0.001 |
| Income (by zip code)  <$38,000  ≥$38,000 | 1,339 (17.4%)  6,359 (82.6%) | 304 (14.1%)  1,852 (85.9%) | <0.001 |
| Education Level (by zip code)  ≥21% with no high school diploma  <21% with no high school diploma | 1,089 (14.1%)  6,609 (85.9%) | 242 (11.2%)  1,914 (88.8%) | 0.001 |
| Population Type  Metropolitan  Urban  Rural | *n = 7,352*  6,006 (81.7%)  1,173 (16.0%)  173 (2.4%) | *n = 2,042*  1,616 (79.1%)  368 (18.0%)  58 (2.8%) | 0.03 |
| Greatest circle distance from treatment center (miles) | 32.8 ± 127.1 | 61.9 ± 173.7 | <0.001 |
| Treatment Center Volume  High (≥3.4 cases/yr)  Low (<3.4 cases/yr) | 1,695 (22.0%)  6,003 (78.0%) | 878 (40.7%)  1,278 (59.3%) | <0.001 |
| Charlson-Deyo Score  0  1  ≥2 | *n = 5,872*  4,315 (73.5%)  1,201 (20.5%)  356 (6.1%) | *n = 1,805*  1,346 (74.6%)  372 (20.6%)  87 (1.8%) | 0.14 |
| Clinical Stage  II  III | 4,049 (52.6%)  3,649 (47.4%) | 1,082 (50.2%)  1,074 (49.8%) | 0.047 |
| T stage (clinical)  T1  T2  T3  T4 | *n = 7,340*  260 (3.6%)  1,976 (26.9%)  4,366 (59.5%)  738 (10.1%) | *n = 2,112*  42 (2.0%)  455 (21.5%)  1,541 (73.0%)  74 (3.5%) | <0.001 |
| N stage (clinical)  N0  N1  N2  N3 | *n = 7,445*  3,244 (43.6%)  3,933 (52.8%)  221 (3.0%)  47 (0.6%) | *n = 2,120*  811 (38.3%)  1,224 (57.7%)  76 (3.6%)  9 (0.4%) | <0.001 |
| Tumor Location (Cervical excluded)  Upper Third  Middle Third  Lower Third | *n = 6,348*  558 (8.8%)  1,422 (22.4%)  4,368 (68.8%) | *n = 1,921*  38 (2.0%)  204 (10.6%)  1,679 (87.4%) | <0.001 |
| Histology  Adenocarcinoma  Squamous Cell Carcinoma | *n = 7,244*  3,947 (54.4%)  3,297 (45.6%) | *n = 1,998*  1,566 (78.4%)  432 (21.6%) | <0.001 |

Supplemental Table 3. Perioperative Outcomes in Patients Undergoing Trimodality Therapy by Age Group

|  | **Overall (n= 2,156)** | **Age 70-79** | **Age ≥80 (n = 145)** | **P Value** |
| --- | --- | --- | --- | --- |
| Length of Hospital Stay (Days) | 14.2 ± 0.33 | 14.3 ± 0.34 | 12.8 ± 1.00 | 0.25 |
| 30-day Readmission | 119 (6.9%) | 111/1597 (7.0%) | 8/119 (6.7%) | 0.90 |
| 30-day Mortality | 106 (5.8%) | 94/1720 (5.5%) | 12/115 (10.4%) | 0.03 |
| 90-day Mortality | 270 (14.8%) | 243/1710 (14.2%) | 27/114 (23.7%) | 0.006 |

Supplemental Table 4. Univariate Analysis of Predictors of Esophagectomy Alone. Within each variable, patients with unknown values were excluded and the number of remaining patients (n) was noted within each variable and treatment group when applicable.

| **Variable** | **Esophagectomy Alone**  **(n=1,215)** | **Concurrent Chemoradiation**  **(n=8,010)** | **P value** |
| --- | --- | --- | --- |
| Age group  70-79 years  ≥80 years | 912 (75.1%)  303 (24.9%) | 5528 (69.0%)  2482 (31.0%) | <0.001 |
| Sex  Male  Female | 940 (77.4%)  275 (22.6%) | 5795 (72.3%)  2215 (27.7%) | <0.001 |
| Race  Caucasian  Non-Caucasian | *n = 1,144*  1139 (95.4%)  55 (4.6%) | *n = 7,952*  7145 (89.9%)  807 (10.1%) | <0.001 |
| Insurance Status  Uninsured  Private  Medicaid  Medicare  Other government | *n = 1,173*  6 (0.5%)  126 (10.7%)  12 (1.0%)  1026 (87.5%)  3 (0.3%) | *n = 7,686*  44 (0.6%)  827 (10.8%)  91 (1.2%)  6611 (86.0%)  113 (1.5%) | 0.017 |
| Income (by zip code)  <$38,000  ≥$38,000 | *n = 1,175*  181 (15.4%)  994 (84.6%) | *n = 7,757*  1360 (17.6%)  6387 (82.4%) | 0.069 |
| Education level (by zip code)  ≥21% with no high school diploma  <21% no high school diploma | *n = 1,175*  143 (12.2%)  1032 (87.8%) | *n = 7,757*  1113 (14.3%)  6644 (85.7%) | 0.045 |
| Population Type  Metropolitan  Urban  Rural | *n = 1,153*  909 (78.8%)  217 (18.8%)  27 (2.3%) | *n = 7,648*  6254 (81.8%)  1210 (15.8%)  184 (2.4%) | 0.036 |
| Greatest circle distance from treatment center (miles) | 54.2 ± 126.9 | 32.7 ± 126.4 | <0.001 |
| Charlson-Deyo Score  0  1  ≥2 | *n = 907*  598 (65.9%)  236 (26.0%)  73 (8.0%) | *n = 6,085*  4473 (73.5%)  1247 (20.5%)  365 (6.0%) | <0.001 |
| Clinical Stage  II  III | 882 (72.6%)  333 (27.4%) | 4193 (52.3%)  3817 (47.7%) | <0.001 |
| Clinical T Stage  Tis/T1  T2  T3  T4 | *n = 1,175*  55 (4.7%)  552 (47.0%)  517 (44.0%)  51 (4.3%) | *n = 7,636*  268 (3.5%)  2051 (21.2%)  4497 (58.9%)  820 (10.7%) | <0.001 |
| Clinical N Stage  N0  N1  N2  N3 | *n = 1,164*  713 (61.3%)  439 (37.7%)  9 (0.8%)  3 (0.3%) | *n = 7,744*  3393 (43.8%)  4070 (52.6%)  233 (3.0%)  48 (0.6%) | <0.001 |
| Tumor location (Cervical excluded)  Upper Third  Middle Third  Lower Third | *n = 1,005*  27 (2.7%)  158 (15.7%)  820 (81.6%) | *n = 6,348*  558 (8.8%)  1422 (22.4%)  4368 (68.8%) | <0.001 |
| Histology  Adenocarcinoma  Squamous cell carcinoma | *n = 1,126*  805 (71.5%)  321 (28.5%) | *n = 7,555*  3960 (52.4%)  3595 (47.6%) | <0.001 |

Supplemental Table 5. Survival After Definitive Therapy Based on Tumor Histology

| **Treatment** | **Histology** | **Median Survival (months)** | **95% CI** | **P Value*** |
| --- | --- | --- | --- | --- |
| Concurrent Chemoradiation | Adenocarcinoma | 14.1 | 13.4 to 14.8 | <0.001 |
|  | Squamous Cell | 14.3 | 13.5 to 15.2 |  |
| Trimodality | Adenocarcinoma | 27.7 | 25.5 to 30.0 | 0.14 |
|  | Squamous Cell | 24.8 | 19.7 to 30.0 |  |

* p values are between histologies within a treatment group
